# 809. Evaluation of Antimicrobial Prophylaxis Prescribing in Post-operative Neurosurgical Procedures

**DOI:** 10.1093/ofid/ofab466.1005

**Published:** 2021-12-04

**Authors:** Clarice Resso, Kelsey Mott, Jasmine Reber, Malika N Kheraj

**Affiliations:** Kaiser Permanente, Philadelphia, PA

## Abstract

**Background:**

Due to lack of relevant clinical data or guidelines, duration of post-operative prophylactic antimicrobial therapy for neurosurgical procedures at the Kaiser Permanente Northern California (KPNC) hospitals has varied among patients and is largely based on the clinical judgment of surgeons. Non-standardized perioperative microbial prophylaxis practice has been associated with excessive antimicrobial therapy, increased risk of drug resistance, institutional cost, and adverse drug effects. This study aims to evaluate differences in prescribing practices of post-operative prophylactic antibiotics among neurosurgeons and to observe the incidence of surgical site infections and adverse drug events caused by antimicrobial prophylaxis that are associated with varying durations of post-procedure prophylaxis.

**Methods:**

A retrospective, observational study of health plan members of KPNC who underwent neurosurgical procedures from 01/01/2011 to 12/31/2019. Prophylactic antimicrobial therapy and duration was identified from medication dispensing and administration records. The study analyzed rates of surgical site infection, post-surgical wound drainage, 60-day mortality, and adverse drug events within 60 days of surgery.

**Results:**

The cohort consisted of 491 patients. 140 received antibiotics for >24 hours and 351 received antibiotics for ≤24 hours. The most common procedures analyzed in our patient population were VP shunt insertion (70.7%) and craniotomy subdural hematoma evacuation (17.9%). There were more surgical site infections in those who received antibiotics >24 hours (4.3% vs 1.4%, p value= 0.05). Univariate logistic regression model showed receiving antibiotics for ≤ 24 hours significantly decreased chance of composite drug event (odds ratio 0.345, 95% CI 0.172-0.691, p value= 0.003).

**Conclusion:**

This study demonstrated that antimicrobial prophylaxis for neurosurgical procedures ≤24 hours decreases risk of adverse drug events. The study provides further evidence to support the development of a standardized protocol that recommends short duration of antibiotic prophylaxis for neurosurgical procedures at KPNC.

Poster

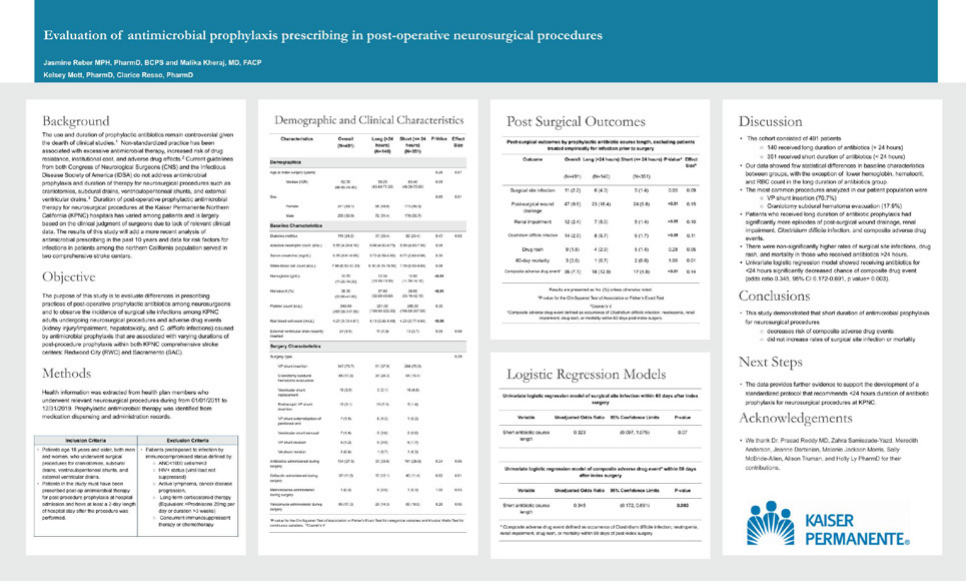

**Disclosures:**

**All Authors**: No reported disclosures

